# Nurturing a Sustainable Transplantation Journey: the best of ESOT Congress 2025

**DOI:** 10.3389/ti.2026.17127

**Published:** 2026-07-14

**Authors:** Olivier Thaunat, Colin Wilson

**Affiliations:** 1 CIRI, Centre International de Recherche en Infectiologie, Université de Lyon, INSERM U1111, Université Claude Bernard Lyon 1, CNRS, UMR5308, ENS de Lyon, Lyon, France; 2 Department of Nephrology, Transplantation and Clinical Immunology, Hospices Civils de Lyon, Groupement Hospitalier Centre, Lyon, France; 3 Lyon-Est Medical Faculty, Claude Bernard University (Lyon 1), Lyon, France; 4 National Institute of Health and Care Research Blood and Transplant Research Unit, Newcastle University and Cambridge University, Newcastle upon Tyne, United Kingdom; 5 Translational and Clinical Research Institute, Newcastle University, Newcastle upon Tyne, United Kingdom; 6 Institute of Transplantation, Freeman Hospital, Newcastle upon Tyne, United Kingdom

## Introduction

This editorial introduces the special issue dedicated to the ESOT London Congress 2025, providing a conceptual and scientific synthesis framed around the “transplantation journey” and the emerging paradigm of sustainable and robust transplantation.

By curating 17 key contributions from the congress, it highlights how recent advances across the entire transplant pathway (from access to long-term graft stewardship) are collectively reshaping contemporary transplant practice.

A central contribution of this congress was the introduction of “robustness” as a solution to improve the sustainability of transplant systems that need not only to be efficient and innovative but also resilient to demographic shifts, biological complexity, and organizational constraints.

Overall, this special issue outlines a forward-looking roadmap for transplantation, bridging technological innovation with responsible implementation and offering a coherent vision for the next era of transplant research and patient care.

## From innovation to responsibility: a new vision for transplantation

The ESOT Congress 2025, held in London from June 29 to July 2, brought together the global transplantation community around a shared vision for the future of the field. The remarkable success of the meeting reflected the enthusiasm of the international community for this ambition. The congress welcomed 3,192 delegates from 91 countries, received 1,655 abstract submissions, generated 989 social media posts using #ESOTCongress, and attracted coverage in more than 1,300 press articles. These figures testify not only to the vitality of transplantation research but also to the growing recognition that transplantation has an important role to play in addressing some of the broader challenges facing modern healthcare systems.

The London congress was organized around a theme that was both timely and transformative: *“Nurturing the Sustainable Transplantation Journey.”* More than a slogan, this theme embodied a collective commitment to reimagining transplantation through the lens of environmental responsibility, and long-term societal impact. The striking animated cover artwork of this special issue captures this vision with remarkable clarity, illustrating the congress philosophy that progress in transplantation should be measured not only by scientific advances but also by our ability to deliver those advances responsibly. However, as highlighted by the Congress Presidents in their welcome address, sustainable transplantation extends far beyond reducing the ecological footprint of healthcare. It encompasses every stage of the transplant pathway—from donor identification and organ procurement to recipient care and lifelong graft stewardship. Central to this vision was the concept of *robustness*, introduced at the congress as the operational counterpart of sustainability. European healthcare systems now prioritize efficiency and productivity-directly eroding human robustness and resilience. A truly sustainable transplantation system must also be a robust one: capable of withstanding disruption, adapting to demographic and epidemiological changes, and delivering reliable outcomes across diverse clinical contexts and healthcare settings. In this perspective, robustness must be a systemic attribute embedded throughout the entire transplantation journey.

**FIGURE 1:**
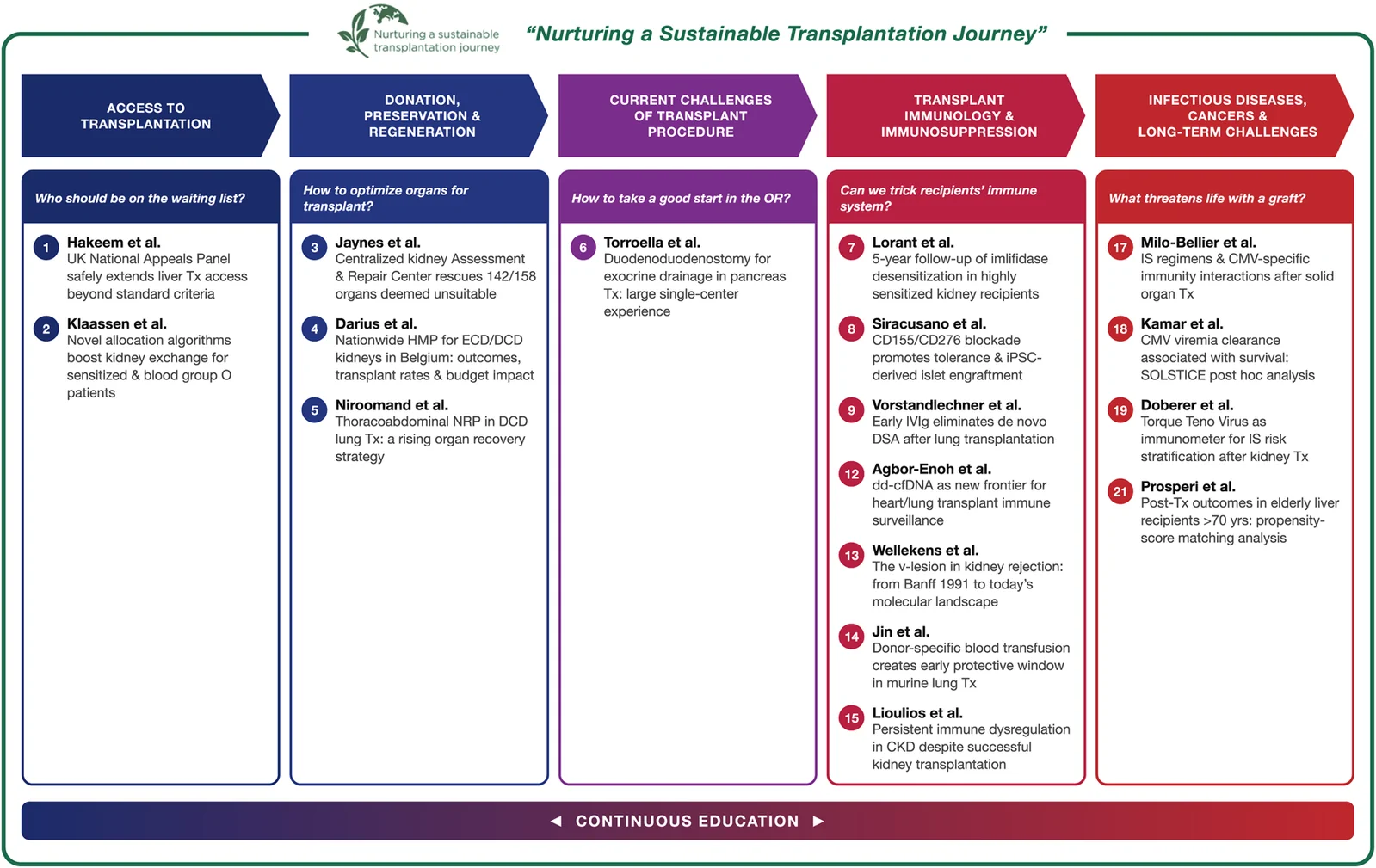
The 17 best abstracts selected from the ESOT Congress 2025 (London, 29 June – 2 July), distributed across the five scientific tracks of the Transplantation Journey. The overarching congress theme—Nurturing a Sustainable Transplantation Journey—Numbers correspond to references cited in the editorial text. References 10–11 (Charmetant et al.) are contextual citations, not congress abstracts. Abbreviations: HMP: hypothermic machine perfusion; NRP: normothermic regional perfusion; DCD: donation after circulatory death; IS: immunosuppression; CMV: cytomegalovirus; DSA: donor-specific antibodies; dd-cfDNA: donor-derived cell-free DNA; CKD: chronickidney disease; Tx: transplantation.

This special issue brings together seventeen outstanding contributions selected from the congress. Organized according to the “Transplantation Journey” framework that structured the scientific programme, these articles illustrate how the field continues to evolve—from expanding access to transplantation and optimizing organ preservation to advancing immunological monitoring and addressing the long-term challenges faced by transplant recipients (Figure 1). Together, they provide a compelling snapshot of a discipline committed not only to innovation, but also to building a more sustainable and robust future for transplantation.

## Expanding access to transplantation

The transplantation journey begins long before surgery. Ensuring equitable access to transplantation remains one of the most important challenges facing the field.

Hakeem et al [1] provide a compelling evaluation of the United Kingdom National Appeals Panel for liver transplantation. Their analysis demonstrates that carefully governed exception pathways can safely extend transplantation to patients who fall outside conventional listing criteria. Despite the complexity and severity of many approved cases, long-term outcomes remained excellent, illustrating how expert review mechanisms can reconcile equity with responsible stewardship of scarce organs.

Similarly, Klaassen et al [2] address another persistent barrier to access: the limited opportunities available to highly sensitized and blood group O patients within kidney exchange programs. Through sophisticated Monte Carlo simulations, they show how innovative allocation algorithms incorporating ABO-incompatible and low-level HLA-incompatible matching can substantially increase transplantation rates for difficult-to-match candidates. Their findings illustrate how intelligent redesign of allocation systems can unlock opportunities without requiring additional donor organs.

Together, these studies remind us that sustainability begins with fairness. Maximizing the benefit derived from every donated organ requires allocation systems capable of adapting to increasingly complex patient populations while maintaining transparency and public trust.

## Optimizing organs: preservation, assessment, and regeneration

The second stage of the transplantation journey focuses on ensuring that every potentially transplantable organ reaches a recipient in optimal condition.

Perhaps no article embodies this philosophy more directly than the report by Jaynes and colleagues [3] describing the first centralized kidney Assessment and Repair Center in the United States. By combining subnormothermic acellular perfusion with centralized viability assessment, the program successfully rescued 142 of 158 kidneys initially considered unsuitable for transplantation. This remarkable achievement demonstrates how novel organizational models may dramatically reduce organ discard while simultaneously improving logistics and organ sharing.

The nationwide Belgian experience reported by Darius and collaborators [4] further illustrates the transformative potential of machine perfusion technologies. Following implementation of a nationally reimbursed hypothermic machine perfusion service, Belgium observed excellent clinical outcomes, increased utilization of DCD kidneys, and substantial healthcare savings. Importantly, the study demonstrates how preservation technologies can generate benefits extending beyond graft outcomes to healthcare system sustainability.

Expanding the donor pool also requires innovation in organ procurement. In their comprehensive review, Niroomand et al [5] examine thoracoabdominal normothermic regional perfusion in donation after circulatory death. By restoring circulation after death determination, this approach mitigates ischemic injury while enabling functional organ assessment. The growing experience with this technique suggests it may become a key component of future strategies aimed at increasing organ availability without compromising quality.

These contributions collectively highlight a major trend emerging across transplantation: the transition from passive preservation toward active organ management. Organs are increasingly viewed not as static biological products but as dynamic systems that can be assessed, optimized, repaired, and even regenerated before transplantation.

## Refining transplantation procedures

Technical excellence remains fundamental to successful transplantation, and advances in surgical practice continue to improve outcomes.

Torroella et al [6] revisit one of the enduring challenges in pancreas transplantation: the optimal method for exocrine drainage. Their large single-center experience with duodenoduodenostomy demonstrates excellent graft and patient survival with acceptable complication rates. Beyond the technical details, this work illustrates how continuous refinement of surgical techniques can incrementally improve outcomes and better reproduce physiological conditions.

Such studies remind us that innovation in transplantation is not limited to breakthrough technologies. Careful reassessment of established practices remains an essential driver of progress.

## Understanding and controlling alloimmunity

The largest group of contributions in this special issue focuses on transplant immunology, reflecting the central role of immune-mediated injury in determining long-term graft survival.

Lorant and colleagues [7] provide encouraging five-year follow-up data on imlifidase-enabled kidney transplantation in highly sensitized recipients. Patients traditionally considered among the most difficult to transplant achieved excellent patient and graft survival despite their extreme immunological risk. These results demonstrate how targeted desensitization strategies can expand access while maintaining favorable long-term outcomes.

At the other end of the spectrum, Siracusano et al [8] offer a glimpse into the future of regenerative transplantation. Using a humanized mouse model, they show that dual blockade of CD155 and CD276 promotes tolerance and facilitates engraftment of iPSC-derived pancreatic islets. As cell therapies move closer to clinical application, such targeted immunomodulatory approaches may become essential for achieving durable graft acceptance without broad immunosuppression.

The complexity of alloimmune responses is further explored through several complementary studies. Vorstandlechner and colleagues [9] demonstrate that early intravenous immunoglobulin therapy may facilitate clearance of donor-specific antibodies after lung transplantation. If these findings are confirmed and are not merely the consequence of the spontaneous disappearance of the dnDSA induced by the inverted direct pathway [10, 11], they would support a proactive intervention before clinical manifestations emerge. Agbor-Enoh and collaborators [12] review the growing role of donor-derived cell-free DNA as a non-invasive biomarker capable of transforming rejection surveillance in thoracic transplantation.

Meanwhile, Wellekens et al [13] revisit one of the most debated lesions in transplant pathology: intimal arteritis. Their historical and contemporary analysis challenges simplistic interpretations of the v-lesion and advocates a more nuanced understanding that incorporates evolving molecular and clinical insights.

Jin et al [14] provide intriguing experimental evidence that donor-specific blood transfusion may transiently reshape the early inflammatory environment after lung transplantation. Although insufficient alone to prevent chronic rejection, this strategy may create a therapeutic window for additional immunomodulatory interventions.

Finally, Lioulios et al [15] remind us that transplantation occurs against a backdrop of profound immune dysregulation associated with chronic kidney disease [16]. Their analysis reveals persistent alterations in regulatory and senescent immune populations even after successful transplantation, highlighting the complexity of immune recovery and adaptation.

Taken together, these studies demonstrate that modern transplant immunology is moving beyond the traditional dichotomy of rejection versus tolerance. Increasingly sophisticated tools are allowing clinicians to characterize, monitor, and manipulate immune responses with unprecedented precision.

## Long-term challenges: infection, monitoring, and aging recipients

The transplantation journey does not end with graft implantation. Long-term success depends on maintaining a delicate balance between immune suppression and immune competence.

Several contributions address cytomegalovirus, one of the most important infectious complications in transplantation. Milo-Bellier et al [17] provide a timely review of the complex interactions between immunosuppressive agents and CMV-specific immunity. Their work emphasizes that infectious risk reflects the cumulative impact of immunosuppression rather than the effects of individual drugs.

Kamar et al [18] extend this discussion through a *post hoc* analysis of the SOLSTICE trial. Their findings demonstrate a striking association between CMV clearance and survival, with no deaths observed among patients achieving viral eradication. These results reinforce the importance of effective antiviral management as a determinant of long-term outcomes.

Doberer et al [19] explore another promising avenue for individualized care through the use of Torque Teno virus as a putative “immunometer” (i.e., biomarker of net immunosuppression [20]). Although challenges remain before widespread implementation, this approach exemplifies the broader movement toward precision immunosuppression guided by objective biological markers.

Finally, Prosperi et al [21] address an increasingly relevant demographic challenge: transplantation in older recipients. Their propensity-matched analysis demonstrates that carefully selected patients over 70 years of age can achieve outcomes comparable to younger recipients. As populations age worldwide, such findings will become increasingly important for ensuring equitable access while maximizing the societal benefits of transplantation.

## Looking forward

The articles collected in this special issue collectively illustrate a field in transition. Across every stage of the transplantation journey, researchers and clinicians are seeking ways to deliver better outcomes while using resources more efficiently, reducing waste, expanding access, and personalizing care.

The concept of sustainable transplantation that defined ESOT 2025 extends far beyond environmental considerations. It encompasses sustainable allocation systems that improve equity, sustainable preservation strategies that reduce organ discard, sustainable immunological monitoring that minimizes unnecessary interventions, and sustainable long-term care that preserves both graft function and patient wellbeing.

The studies highlighted here demonstrate that these goals are no longer aspirational. They are already shaping clinical practice and research priorities across the transplantation community.

As transplantation enters a new era characterized by machine perfusion, regenerative medicine, precision immunology, and data-driven decision making, the challenge will be to ensure that innovation remains aligned with responsibility. The success of ESOT 2025 suggests that the community is ready to embrace this challenge.

The future of transplantation will not be defined solely by what we can achieve scientifically, but by how wisely, equitably, and sustainably we apply those achievements for the benefit of patients worldwide. This special issue offers a glimpse of that future—and it is an inspiring one.

## Group members of scientific program committee

Anya Adair (United Kingdom), Varuna Aluvihare (United Kingdom), Oriol Bestard (Spain), Ekaterine Berishvili (Switzerland), Gregor Bond (Austria), Saskia Bos (United Kingdom), Philippe Compagnon (Switzerland), Lionel Couzi (France), Lucy Dames (United Kingdom), Caroline Dudreuilh (United Kingdom), Giuseppe Feltrin (Italy), Hermien Hartog (Netherlands), Speranta Iacob (Romania), Sophie Limou (France), Sandra Lindstedt (Sweden), Maarten Naesens (Belgium), Gianluca Rompianesi (Italy), Jelena Stojanovic (United Kingdom), Olivier Thaunat (ESOT President, Co-Chair, France), Fabio Vistoli (Italy), Robin Vos (Belgium), Colin Wilson (Co-Chair, United Kingdom).

## References

[1] Hakeem AR Gupta S Taylor R Grammatikopoulos T Masson S Prasad R The UK national appeals panel safely extends access to liver transplantation for candidates beyond standard listing criteria. Transpl Int (2026) 39:15573. 10.3389/ti.2026.15573 41675921 PMC12886060

[2] Klaassen MF De Klerk M Baas MC Bouwsma H Bungener LB Christiaans MHL Novel allocation strategies can boost kidney exchange programs: a monte carlo simulation. Transpl Int (2026) 39:15423. 10.3389/ti.2026.15423 41858495 PMC12995821

[3] Jaynes CL Goggins WC Holzner ML Garonzik-Wang J Leuvenink HGD . No kidney left behind: rescuing unused donor kidneys for transplant at the first centralized assessment and repair center. Transpl Int (2025) 38:15424. 10.3389/ti.2025.15424 41394518 PMC12698490

[4] Darius T Jochmans I Foguenne M Hoste E Randon C Bracke B Nationwide hypothermic machine perfusion for ECD and DCD kidney transplantation in Belgium: one-year outcomes and impact on transplant rates and budget impact analysis. Transpl Int (2025) 38:15282. 10.3389/ti.2025.15282 41323959 PMC12657249

[5] Niroomand A Chang S Lindstedt S . Back in circulation: a review of the implementation of thoracoabdominal normothermic regional perfusion in donation after circulatory death in lung transplantation. Transpl Int (2026) 39:15272. 10.3389/ti.2026.15272 42130672 PMC13160682

[6] Torroella A Zhu RH Castillo-Delgado C Pavesi M Rull R Folch-Puy E Duodenoduodenostomy as an attractive option for exocrine drainage in pancreas transplantation: insights from a single-center cohort. Transpl Int (2025) 38:15430. 10.3389/ti.2025.15430 41256736 PMC12620303

[7] Lorant T Lonze BE Montgomery RA Desai NM Legendre C Lundgren T Five years Follow-up of imlifidase desensitized kidney transplant recipients. Transpl Int (2025) 38:15425. 10.3389/ti.2025.15425 41394519 PMC12697064

[8] Siracusano G Deambrogio F Sordi V Malnati M Piemonti L Chimienti R . Blockade of CD155 and CD276 by monoclonal antibodies fosters immune tolerance and promotes stable engraftment of iPSC-Derived islets in allogeneic humanized mice. Transpl Int (2025) 38:15433. 10.3389/ti.2025.15433 41403780 PMC12702790

[9] Vorstandlechner M Degenfelder P Yavuz G Glueck OM Kovács JR Walter J Efficacy of Intravenous Immunoglobulin in Eliminating *de novo* Donor-Specific Antibodies After Lung Transplantation: importance of Early Intervention. Transpl Int (2025) 38:15350. 10.3389/ti.2025.15350 41256738 PMC12620304

[10] Charmetant X Pettigrew GJ Thaunat O . Allorecognition unveiled: integrating recent breakthroughs into the current paradigm. Transpl Int (2024) 37:13523. 10.3389/ti.2024.13523 39588197 PMC11586167

[11] Charmetant X Chen CC Hamada S Goncalves D Saison C Rabeyrin M Inverted direct allorecognition triggers early donor-specific antibody responses after transplantation. Sci Transl Med (2022) 14(663):eabg1046. 10.1126/scitranslmed.abg1046 36130013

[12] Agbor-Enoh S Fraser E Nadella N Andargie TE Alnababteh M . dd-cfDNA: the new frontier for heart/lung transplant surveillance? Transpl Int (2025) 38:15555. 10.3389/ti.2025.15555 41531497 PMC12791046

[13] Wellekens K Koshy P Roufosse C Naesens M . From banff 1991 to today: the changing landscape of the v-Lesion in kidney transplant rejection. Transpl Int (2025) 38:14818. 10.3389/ti.2025.14818 40661921 PMC12256300

[14] Jin X Hooft C Özsoy B Van Slambrouck J Kaes J Vanluyten C Donor-specific blood transfusion induces a transfusion-related early protective effect in murine lung transplantation. Transpl Int (2026) 39:15409. 10.3389/ti.2026.15409 42147823 PMC13171468

[15] Lioulios G Moisidou E Christodoulou M Kasimatis E Xochelli A Memmos E Cellular immunity in chronic kidney disease and changes after kidney transplantation. Transpl Int (2026) 39:15622. 10.3389/ti.2026.15622 41635523 PMC12862250

[16] Espi M Charmetant x Fusil F Mathieu C Legras M Pelletier C Chronic Kidney Disease-Associated Defect in Humoral Immune Response Is Driven by Inflammation. Toxins (2026) 18 (2):104. 10.3390/toxins18020104 41745770 PMC12944840

[17] Bellier LM Kaminski H Merville P Couzi L . Interactions between immunosuppressive regimens and cytomegalovirus infection after solid-organ transplantation. Transpl Int (2026) 39:15987. 10.3389/ti.2026.15987 42040200 PMC13102634

[18] Kamar N Avery RK Bo T Gu J Kumar D Witzke O . Association between cytomegalovirus viremia clearance and post-solid organ transplant mortality in patients with refractory cytomegalovirus infection: SOLSTICE post hoc analysis. Transpl Int (2025) 38:15331. 10.3389/ti.2025.15331 41384277 PMC12689443

[19] Doberer K Kapps S Haupenthal F Bond G . Immune monitoring goes viral – torque teno virus for immunologic risk stratification after kidney transplantation. Transpl Int (2025) 38:15074. 10.3389/ti.2025.15074 41323957 PMC12660151

[20] Chauvelot L Barba T Saison C Siska E Kulifaj D Bakker S. J. L. Longitudinal monitoring of torque teno virus DNAemia in kidney transplant recipients correlates with long-term complications of inadequate immunosuppression. J. Med. Virol. (2024) 96 (e29806). 10.1002/jmv.29806 39007420

[21] Prosperi E Prosperi E Serenari M Bonatti C Fallani G Stocco A Evaluating post-transplant outcomes in elderly liver recipients over 70: a propensity-score matching analysis. Transpl Int (2025) 38:15429. 10.3389/ti.2025.15429 41368131 PMC12682703

