# Correction: Nurturing a sustainable transplantation journey: the best of ESOT congress 2025

**DOI:** 10.3389/ti.2026.17532

**Published:** 2026-08-28

**Authors:** Transplant International Production Office

**Affiliations:** Frontiers Media SA, Lausanne, Switzerland

There was a mistake in Figure 1 as published. The citations for references Milo-Bellier et al, Kamar et al, and Prosperi et al. were included incorrectly. The corrected Figure 1 appears below.

**FIGURE 1:**
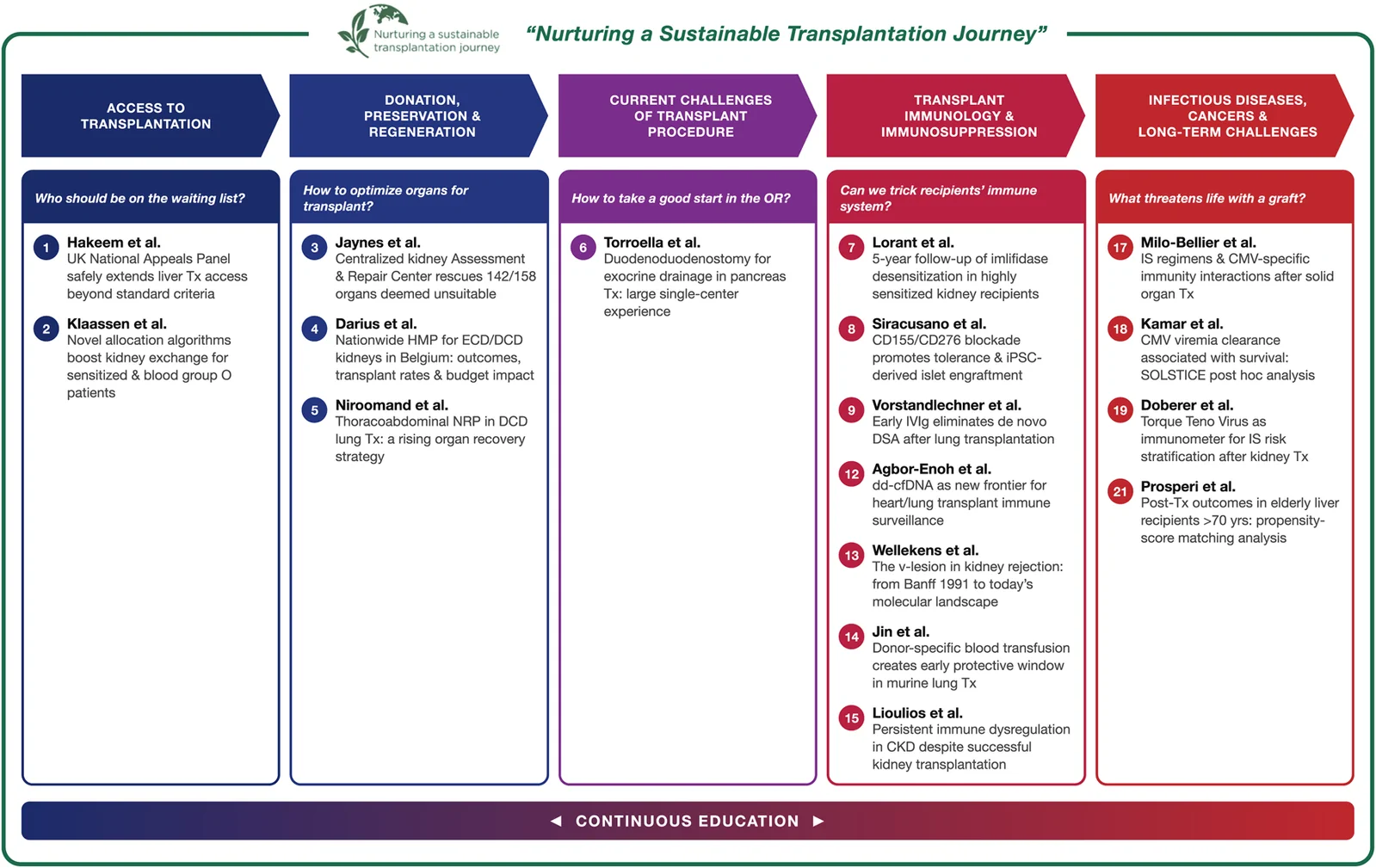
The 17 best abstracts selected from the ESOT Congress 2025 (London, 29 June – 2 July), distributed across the five scientific tracks of the Transplantation Journey. The overarching congress theme —Nurturing a Sustainable Transplantation Journey —Numbers correspond to references cited in the editorial text. References 10–11 (Charmetant et al.) are contextual citations, not congress abstracts. Abbreviations: HMP: hypothermic machine perfusion; NRP: normothermic regional perfusion; DCD: donation after circulatory death; IS: immunosuppression; CMV: cytomegalovirus; DSA: donor-specific antibodies; dd-cfDNA: donor-derived cell-free DNA; CKD: chronickidney disease; Tx: transplantation.

The original version of this article has been updated.

## Generative AI statement

Any alternative text (alt text) provided alongside figures in this article has been generated by Frontiers with the support of artificial intelligence and reasonable efforts have been made to ensure accuracy, including review by the authors wherever possible. If you identify any issues, please contact us.

